# Efficacy and safety of sofosbuvir plus daclatasvir in hemodialysis patients with genotype 1b or 2a hepatitis C virus infection: a single-arm, prospective real-world study

**DOI:** 10.3389/fmed.2025.1576654

**Published:** 2025-07-09

**Authors:** Kaili Wang, Hongyu Yao, Xia Zhou, Hongling Liu, Jun Zhao

**Affiliations:** Senior Department of Hepatology, Fifth Medical Center of Chinese PLA General Hospital, Beijing, China

**Keywords:** daclatasvir, sofosbuvir, chronic hepatitis C virus, hemodialysis, sustained virologic response, direct-acting antiviral agents

## Abstract

**Background/aims:**

This investigation assessed the clinical outcomes and adverse effects of combination therapy using sofosbuvir (SOF) and daclatasvir (DCV) among dialysis-dependent patients infected with hepatitis C virus (HCV) genotypes 1b or 2a in real-world settings.

**Methods:**

We conducted a prospective, single-arm interventional trial comprising 16maintenance hemodialysis patients (14 with HCV-1b, 2 with HCV-2a). Participants received SOF-DCV combination therapy over 24 weeks with monitoring at weeks 4, 12, and 24 during treatment, plus a follow-up assessment 12 weeks post-treatment completion. The primary outcome measure was sustained virologic response at 12 weeks post-treatment (SVR12). Secondary endpoints included therapeutic tolerance and safety profiles.

**Results:**

All 16 participants completed the prescribed treatment regimen. Demographic characteristics revealed a mean age of 57.0 years, male predominance (75%), average dialysis duration of 7.0 years, and mean body weight of 63.0 kg. Five patients (31.3%) had compensated cirrhosis. Liver function parameters remained stable throughout the study period. Rapid virologic response (RVR) was documented in 87.5% (14/16) of participants, while end-of-treatment response (ETR) and SVR12 were both achieved in 93.8% (15/16) of cases. All cirrhotic patients (5/5) ultimately attained SVR12. The therapeutic regimen demonstrated favorable tolerability, with no treatment discontinuations due to adverse events. One participant was lost to follow-up. APRI scores significantly decreased from baseline (0.56) to week 24 (0.20, *p* < 0.001). Reported adverse reactions included headache, fatigue, nausea (each 6.3%), and anemia (18.8%).

**Conclusion:**

The 12-week SOF-DCV combination demonstrated robust therapeutic efficacy and acceptable safety profiles in hemodialysis patients infected with HCV genotypes 1b or 2a, including those with compensated cirrhosis.

## 1 Background

Chronic hepatitis C virus (HCV) represents a significant global health burden, particularly among individuals receiving maintenance hemodialysis (MHD). The infection rate varies considerably across regions, affecting between 6 and 44% of MHD patients worldwide (1, 2). This patient population faces heightened risks of adverse outcomes, including accelerated progression to cirrhosis, development of hepatocellular carcinoma (HCC), and increased liver-related mortality, underscoring the critical importance of therapeutic intervention (3, 4). The therapeutic landscape for HCV has evolved dramatically over time. Traditional treatment approaches relied on pegylated-interferon (peg-IFN), either as monotherapy or combined with ribavirin (RBV). However, these protocols were hampered by numerous limitations, including extended treatment periods, suboptimal viral clearance rates, poor patient tolerance, and substantial adverse effects (5, 6).

The introduction of direct-acting antivirals (DAAs) marked a revolutionary advancement in HCV treatment, achieving sustained virological response (SVR) rates exceeding 95% (7). These agents specifically target crucial phases of viral replication, representing a significant therapeutic breakthrough. Despite their remarkable success in the general population, comprehensive data regarding their efficacy and safety profile in hemodialysis patients remains limited. The combination of sofosbuvir (SOF) and daclatasvir (DCV), with or without ribavirin supplementation, has demonstrated exceptional effectiveness against HCV infection, regardless of cirrhotic status (8). However, SOF presents unique considerations in renal impairment, as its active metabolite undergoes renal elimination. Elevated SOF levels in severe renal dysfunction have raised theoretical concerns about potential cardiovascular and hepatobiliary complications, though human toxicity data remains inconclusive. Current guidelines restrict SOF-based regimens to patients with estimated glomerular filtration rates (eGFR) ≥ 30 mL/min per 1.73m^2^. Nevertheless, real-world evidence from hemodialysis patients receiving SOF-based treatments has not identified significant safety concerns (9).

DCV offers potential advantages for patients with severe renal impairment due to its predominant hepatic metabolism. However, contemporary HCV treatment guidelines lack comprehensive data regarding the safety and efficacy of DAA combinations, particularly SOF plus DCV, in the hemodialysis population. This knowledge gap prompted our investigation into the therapeutic outcomes and safety profile of SOF-DCV combination therapy in HCV-infected hemodialysis patients.

## 2 Patients and methods

### 2.1 Patients

A prospective observational trial was initiated at the Fifth Medical Center of Chinese PLA General Hospital, spanning from May 2017 through May 2019. The therapeutic protocol involved administering SOF (400 mg thrice weekly) alongside daily DCV (60 mg) to 16HCV-positive dialysis patients over a 12-week duration. Study participation required meeting these essential criteria: (1) individuals aged 18 or above, encompassing males and females (excluding pregnancy/lactation); (2) verified HCV chronicity through RNA detection; (3) current dialysis therapy; (4) liver function without decompensation indicators; (5) DAA treatment-naïve statusCirrhosis was diagnosed based on a combination of clinical, laboratory, and imaging findings, including FibroScan results >12 kPa, characteristic ultrasonographic features, and/or AST-to-platelet ratio index (APRI) values >1.5. All cirrhotic patients included in this study had compensated cirrhosis with Child-Pugh class A status, without any signs of decompensation such as ascites, encephalopathy, or variceal complications.

Participants were excluded based on: (1) non-HCV hepatic disorders or HIV/HBV presence; (2) existing or suspected malignancies, including hepatocellular carcinoma; (3) signs of liver decompensation (encompassing ascites, encephalopathy, variceal complications); (4) transplant history involving organs or bone marrow; (5) major organ system dysfunction or inadequately managed diabetes/hypertension; (6) ongoing use of immune suppressants, experimental compounds, or agents impacting renal function; (7) known allergies to treatment compounds; and (8) current substance misuse.

All patients underwent standard thrice-weekly hemodialysis sessions (4 h each) throughout the study period, with medication administration scheduled after dialysis sessions to minimize potential drug removal. Formal written consent was obtained from all participants.

For patients with cirrhosis, additional cardiac evaluation was performed at baseline, including echocardiography to assess for cirrhotic cardiomyopathy. Monitoring of cardiovascular parameters continued throughout the treatment period.

### 2.2 Measurements

Viral load quantification was performed using COBAS TaqMan HCV v2.0 PCR assays (Roche Molecular Diagnostics, Pleasanton, CA, USA). Sample analysis followed the intention-to-treat methodology. The AST-to-platelet ratio index (APRI) was determined using the formula: APRI = ([AST/ULN]/PLT) × 100, where AST ULN = 40 U/L (10).

### 2.3 Outcomes

Study evaluation centered on two key metrics: SVR12 attainment and therapeutic safety profile. Treatment success was defined by viral RNA suppression below measurable levels (15 IU/mL) at the 12-week post-therapy mark. Patient monitoring encompassed documentation of adverse reactions (including hematologic and cutaneous manifestations) and therapy cessation incidents. Additional outcome measures tracked viral response dynamics (weeks 4, 8, 12), liver enzyme normalization patterns, and treatment failures - including viral rebound and post-treatment recurrence. Protocol mandated therapy termination upon viral reactivation, specifically when RNA levels surpassed 10^2^ IU/mL following initial viral clearance.

### 2.4 Statistical analysis

Statistical evaluation utilized SPSS software (version 20). Data representation followed standard conventions: categorical variables as proportional distributions, numeric data as mean values with standard deviations. Comparative analyses incorporated both independent and matched t-testing methodologies. APRI progression underwent polynomial-based ANOVA examination. Results achieving *p*-values below 0.05 were considered statistically significant.

## 3 Results (revised)

### 3.1 Baseline characteristics

The study enrolled sixteen hemodialysis patients with HCV infection. The demographic and clinical characteristics are presented in Table 1. The patient population consisted predominantly of genotype 1b HCV infections (14 patients, 87.5%), with the remaining cases being genotype 2a (2 patients, 12.5%). Participants had a median age of 57.0 years, with male predominance (75%, *n* = 12). The average dialysis duration was 7.0 years.

**Table 1:** Baseline characteristics of study participants (*n* = 16).

Characteristics	Value
Age (Years), median (range)	57 (35–71)
Male/female, *n*	12/4
Weight (kg), median (range)	63 (53.4–73.2)
Duration of Dialysis (Years), median (range)	7 (2–16)
Liver cirrhosis, *n* (%)	5 (31.3)
HCV RNA genotype, *n* (%)	
1b	14 (87.5)
2a	2 (12.5)
HCV RNA, log10 IU/mL, median	5.7
White Blood Cells, ×10^3^/mm^3^, median	4.5
Hemoglobin (g/L), median	109.5
Platelets, ×10^3^/mm^3^, median	90
ALT (IU/L), median	15.5
AST (IU/L), median	15
Creatinine (μmol/L), median	769
Comorbidities, *n* (%)	
Diabetes mellitus	14 (87.5)
Hypertension	12 (75)
APRI, median	0.56
Prior HCV treatment, *n* (%)	
Treatment-naïve	13 (81.3)
Peg-IFN experienced	3 (18.7)
DAA-naïve	16 (100)
Cardiac status in cirrhotic patients (*n* = 5)	
Left ventricular ejection fraction (%), median (range)	58 (52–64)
Evidence of cardiomyopathy, *n*	0
Characteristics	Value

ALT, alanine aminotransferase; AST, aspartate aminotransferase; APRI, AST-to-platelet ratio index; DAA, direct-acting antiviral; HCV, hepatitis C virus; Peg-IFN, pegylated interferon.

Liver status assessment revealed compensated cirrhosis (Child-Pugh A) in five patients (31.3%), while others presented with chronic hepatitis. Baseline cardiac evaluation of cirrhotic patients showed no significant abnormalities, with all patients having preserved ejection fraction (>50%) and no evidence of cirrhotic cardiomyopathy. The underlying renal dysfunction was primarily attributed to diabetes mellitus (DM; 87.5%, 14/16) and hypertension (75%, 12/16).

Regarding prior HCV treatment, three patients had received pegylated interferon-based therapy but had not achieved SVR, while the remainder were treatment-naïve. All patients were DAA-naïve at enrollment.

### 3.2 Efficacy

#### 3.2.1 Virologic response

Rapid virologic response (RVR), defined as undetectable HCV RNA after 4 weeks of therapy, was achieved in 14/16 patients (87.5%). End of treatment response (ETR) showed undetectable HCV RNA in 15/16 patients (93.8%). Sustained virologic response (SVR) at 12 weeks post-treatment was maintained in 15/16 patients (93.8%). When analyzed by genotype, SVR12 was achieved in 13/14 (92.9%) patients with genotype 1b and 2/2 (100%) patients with genotype 2a. Among cirrhotic patients, one failed to achieve RVR; however, all cirrhotic patients ultimately achieved both ETR and SVR12. One patient who initially achieved RVR was lost to follow-up despite demonstrating good treatment tolerance (Table 2).

**Table 2:** Virologic response by intention-to-treat analysis (*n* = 16).

Virologic response	No. (%)	Genotype 1b (*n* = 14)	Genotype 2a (*n* = 2)	Cirrhosis (*n* = 5)	No cirrhosis (*n* = 11)
Week 4 (RVR)	14 (87.5)	12 (85.7)	2 (100)	4 (80)	10 (90.9)
Week 8	14 (87.5)	12 (85.7)	2 (100)	4 (80)	10 (90.9)
Week 12 (ETR)	15 (93.8)	13 (92.9)	2 (100)	5 (100)	10 (90.9)
Week 24 (SVR12)	15 (93.8)	13 (92.9)	2 (100)	5 (100)	10 (90.9)
Virologic breakthrough	0 (0)	0 (0)	0 (0)	0 (0)	0 (0)
Viral relapse	0 (0)	0 (0)	0 (0)	0 (0)	0 (0)
Lost to follow-up	1 (6.3)	1 (7.1)	0 (0)	0 (0)	1 (9.1)

RVR, rapid virologic response; ETR, end of treatment response; SVR12, sustained virologic response at 12 weeks post-treatment.

#### 3.2.2 ALT normalization

Pre-treatment baseline values for ALT and AST were 15.5 U/L and 15 U/L, respectively. Throughout the study period, no instances of ALT or AST elevation were observed. Complete blood indices remained stable, showing no significant variations from baseline through week 24 across all groups (Table 3).

**Table 3:** Clinical and laboratory parameters at week 24.

Characteristics	Value (*n* = 15)	*p*-value (vs. baseline)
White Blood Cells, ×10^3^/mm^3^, median	5.3	0.07
Hemoglobin (g/L), median	119	0.04*
Platelets, ×10^3^/mm^3^, median	134	0.02*
ALT (IU/L), median	11.5	0.09
AST (IU/L), median	16.0	0.22
Creatinine (μmol/L), median	833	0.13
APRI, median	0.20	<0.001*

ALT, alanine aminotransferase; AST, aspartate aminotransferase; APRI, AST-to-platelet ratio index. Statistically significant (^*^p < 0.05).

#### 3.2.3 Non-invasive liver fibrosis measurements

**Figure 1:**
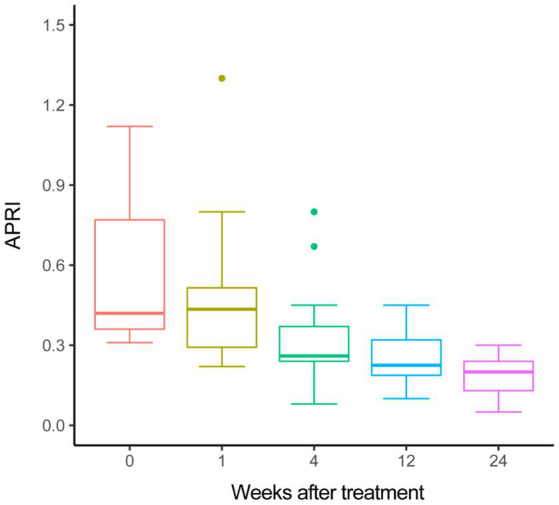
APRI changes during treatment.

Furthermore, we assessed the change of liver fibrosis by non-invasive measurement. The median value of APRI significantly decreased from 0.56 at treatment baseline to 0.20 at week 24 by use of one-way ANOVA (*p* < 0.001), moreover, the decline trend of APRI during the treatment period was statistically significant by use of a polynomial contract procedure of ANOVA (*p* < 0.001, Figure 1).

### 3.3 Safety

All patients (100%) maintained their regular hemodialysis schedule throughout the study period without interruptions. A total of 15 patients (93.8%) completed 12 weeks of therapy and the follow-up period. The majority of adverse events were of mild or moderate intensity. No drug discontinuation occurred owing to adverse events over the whole follow-up period. The most prevalent symptoms were headache (6.3%), fatigue (6.3%), nausea (6.3%), and anemia (18.8%). No episodes of elevated ALT or total bilirubin occurred during the study period. Anemia was observed in 3 patients (18.8%), possibly attributable to preexisting renal illness (Table 4). No cardiovascular complications were observed in patients with cirrhosis.

**Table 4:** Adverse events and laboratory abnormalities.

Sofosbuvir/daclatasvir for 12 weeks (*n* = 16)	*n* (%)
Any adverse events	5 (31.3)
Serious adverse events	0 (0)
Most common adverse events	
Anemia	3 (18.8)
Headache	1 (6.3)
Fatigue	1 (6.3)
Nausea	1 (6.3)
Thrombocytopenia	1 (6.3)
Cardiac events in cirrhotic patients (*n* = 5)	0 (0)

Some patients experienced more than one adverse event.

## 4 Discussion

The landscape of HCV treatment in hemodialysis patients has dramatically evolved from the interferon era, which was characterized by poor tolerance and suboptimal SVR rates. The emergence of direct-acting antivirals (DAAs) has transformed therapeutic outcomes, offering superior SVR rates with improved tolerability and reduced toxicity.

Our investigation, involving 16 hemodialysis patients with HCV genotypes 1b and 2a, demonstrated impressive clinical outcomes with a 12-week SOF-DCV regimen. The achievement of both ETR and SVR in 93.8% (15/16) of participants aligns with previously documented success rates in clinical trials and real-world studies of SOF-based therapies (9–12). These findings reinforce SOF-based combinations, particularly SOF plus DCV, as a preferred therapeutic approach for HCV genotypes 1b and 2a in this population.

Recent meta-analyses examining DAA efficacy in maintenance hemodialysis patients have reported SVR rates ranging from 66.7 to 98.3%, with SOF-based regimens specifically achieving 89.4% SVR (13, 14). Our observed SVR rate of 93.8% in the intention-to-treat population corroborates these findings. Chronic HCV infection is associated with progressive liver complications, including fibrosis, hepatic failure, and hepatocellular carcinoma (HCC). The advent of DAAs, achieving SVR rates approaching 90% in clinical practice, has revolutionized disease management. Successful viral eradication through SVR achievement can halt or reverse hepatic damage, thereby reducing liver-related complications. Supporting evidence comes from an 18-month investigation that evaluated post-DAA fibrosis changes using ARFI measurements, demonstrating significant improvement in liver stiffness among patients achieving SVR at 24 weeks post-treatment (15).

Recent work (16) has highlighted the complex interaction between the human microbiota, immune system, and HCV infection, suggesting potential additional mechanisms through which DAA therapy may exert beneficial effects beyond direct viral suppression. This emerging perspective offers new insights into the comprehensive benefits of DAA therapy in special populations such as hemodialysis patients. The high tolerability of SOF observed in previous studies was replicated in our investigation, with only a single patient lost to follow-up. Our study population’s genotype distribution mirrors that reported in meta-analyses, with HCV genotype 1b predominating (14 patients, 87.5%), followed by genotype 2a (2 patients, 12.5%). Notably, neither SOF nor its inactive metabolites showed evidence of accumulation, regardless of hemodialysis timing or treatment duration. The regimen demonstrated a favorable safety profile with no serious adverse events reported, including no cardiovascular complications in cirrhotic patients. Our findings align with other investigations demonstrating that SOF-based regimens maintain both efficacy and tolerability in end-stage renal disease (ESRD) patients on maintenance hemodialysis, despite their severely reduced eGFR (<10 mL/min/1.73m^2^) (17, 18). The superior SVR rates achieved with our modified SOF dosing regimen (400 mg thrice weekly) suggest this approach as a viable treatment strategy for this population. However, larger-scale investigations are particularly crucial in developing nations where HCV prevalence among maintenance hemodialysis patients remains elevated.

HCV infection maintains endemic status in China and other developing nations, with projections indicating an increasing burden among hemodialysis patients, primarily due to inadequate medical safety practices. Our results demonstrate that SOF combined with DCV offers a highly effective and well-tolerated treatment option for ESRD patients with genotypes 1b and 2a undergoing maintenance hemodialysis. Given SOF’s position as a cornerstone of DAA therapy in developing regions, attributed to both its efficacy and cost-effectiveness, expanded clinical investigations are essential to further validate these findings. While the findings are intriguing, the small sample size and short follow-up period collectively limit the generalizability of the results in this study. The long-term evolution post-DAA treatment remains insufficiently evaluated, necessitating further studies to explore the impact of HCV infection and DAA therapy on risk of cirrhosis decompensation and oncogenesis.

## Data Availability

The original contributions presented in the study are included in the article/Supplementary material, further inquiries can be directed to the corresponding authors.
